# Evidence of insecticide resistance in *Aedes aegypti* populations of Suriname: first report of malathion resistance and presence of the *kdr* mutations V410L, V1016I and F1534C

**DOI:** 10.1590/0074-02760250335

**Published:** 2026-07-10

**Authors:** Kartika Doerdjan-Ramoutar, Jerry Toelsie, Amandine Guidez, Treyanti Soekhoe, Jean-Bernard Duchemin

**Affiliations:** 1 Anton de Kom University of Suriname, Faculty of Mathematics and Natural Sciences, Department Biology, Paramaribo, Suriname; 2 Anton de Kom University of Suriname, Faculty of Medical Sciences, Department Physiology, Paramaribo, Suriname; 3 Pasteur Institute, Cayenne, French Guiana; 4 Bureau for Public Health, Suriname Department Entomology, Paramaribo, Suriname

**Keywords:** Aedes aegypti, insecticide resistance, *kdr* mutation, malathion, λ-cyhalothrin

## Abstract

**BACKGROUND:**

Dengue outbreaks pose a significant public health risk in Suriname, with challenges in mosquito surveillance and limited data on insecticide resistance hampering control efforts.

**OBJECTIVES:**

To investigate the resistance status and involved mechanisms of *Aedes aegypti* mosquitoes for malathion and λ-cyhalothrin insecticides.

**METHODS:**

The Centres for Disease Control and Prevention (CDC) bottle bioassay was used to test the resistance phenotypic status of *Ae. aegypti* while the occurrence and frequency of knockdown resistance (*kdr*) mutations were accessed by TaqMan genotype assays for the sites 410L (Valine/Leucine), 1016I (Valine/Isoleucine), and 1534C (Phenylalanine/Cysteine).

**FINDINGS:**

Results showed resistance to malathion in the Blauwgrond population, with other regions exhibiting reduced susceptibility based on mortality rates between 89% and 94%. Very low mortality rates indicate resistance to λ-cyhalothrin in all tested areas. Knockdown resistance (*kdr*) mutations were detected at high frequencies. The triple homozygous *kdr* genotype leucine/leucine, isoleucine/isoleucine, cysteine/cysteine (LL/II/CC) predominated (84.2%), while the wild-type genotype was found only in 1.8% of the samples.

**MAIN CONCLUSION:**

This study reports the first detection of malathion resistance in *Ae. aegypti* from Suriname and confirms high levels of resistance to λ-cyhalothrin, possibly driven by *kdr* gene mutations. The results emphasise the importance of sustained surveillance and continued research on resistance mechanisms to guide effective and evidence-based vector control strategies.

*Aedes aegypti* (Diptera: Culicidae) is a major threat to public health in the tropics and subtropics because it transmits arboviruses such as dengue, Zika, chikungunya, and yellow fever. Recent data from the Pan American Health Organization (PAHO) mentioned a serious increase in severe dengue cases in the past decades in the region of the Americas, with a record of 4,565,911 reported infections, including 7653 severe cases and 2340 deaths in 2023.^1^ In Suriname, a middle-income country located in the north of South America, regular outbreaks of dengue are reported, and this country was confronted with the first outbreak of chikungunya in June 2014.^2^ Since 2015, Zika infections have unfortunately also been detected in Suriname with an outbreak in 2016.^3^ Vector control, such as insecticide spraying, is still the most used method to prevent infections.^4^ [Bibr B5] ^5^ However, a potential threat that undermines the success of vector control programs is the development of resistance to insecticides in the vector.

Many studies from different parts of the world, including the Caribbean and Latin America, have shown that *Ae. aegypti* develop resistance to commonly used insecticides^5^ [Bibr B6] ^6^ such as Organophosphates, including malathion, fenitrothion, and Pyrethroids such as deltamethrin and cypermethrin.^5^ [Bibr B7] ^7^ [Bibr B8] ^8^ Remarkably, resistance to various insecticides may vary in each country or even area.^9^ [Bibr B10] ^10^ This makes it necessary for every country touched by mosquito-borne diseases, especially in the low and middle-income countries, to conduct regular surveys of resistance prevalence within the vector population with dynamics between areas.

Four known mechanisms may be associated with insecticide resistance in arthropods, namely: metabolic mechanisms, target-site resistance, reduction of cuticle penetration, and behavioural change. The first two mechanisms are the most common and widely described. Metabolic mechanisms involve changes in enzyme activities in the mosquito that cause rapid insecticide detoxification and prevent the active ingredients from reaching their target. Enzymes involved are especially those from the classes esterases, monooxygenases, and glutathione S-transferases. Synergists are chemicals that inhibit these enzymes in mosquitoes and are used in combination with insecticides to enhance their potency. Target-site resistance involves changes in certain specific protein receptors originally targeted by the insecticide as a result of gene mutations. Known are, for example, mutations known as knockdown resistance (*kdr*), which reduce the effectiveness of pyrethroids by affecting their target site, the voltage-gated sodium channel.^11^ [Bibr B7] ^7^ [Bibr B12] ^12^

Exposure to insecticides occurs in various ways, such as through government-organised control campaigns, or in agricultural areas, or simply through domestic use in households. At the national level in Suriname, control campaigns are being carried out, and areas with a high transmission risk have been identified.^13^ From the 1970s, control measures included spraying with insecticides such as malathion, fenithrothion, and, since 2019, λ-cyhalothrin, known as karatox. As a form of biological control, the Bti (*Bacillus thuringiensis israelensis*) formulation was introduced in 2011 for larval control.^13^

The Bureau for Public Health in Suriname (BOG) reported reduced susceptibility to deltamethrin and permethrin (BOG unpublished data), while a study in 2017 suggested a possible resistance to malathion in mosquito samples from the area Blauwgrond.^14^ No studies have been conducted in recent years on the susceptibility status of *Ae. aegypti*, and any mechanisms responsible for resistance in mosquitoes have not been previously investigated in Suriname.

Therefore, in this study, we assessed the phenotype and genotype insecticide resistance status of the *Ae. aegypti* field population against the commonly used organophosphate malathion and the pyrethroid λ-cyhalothrin in several districts of Suriname where regular dengue outbreaks are reported.

## MATERIALS AND METHODS

**Fig. 1:**
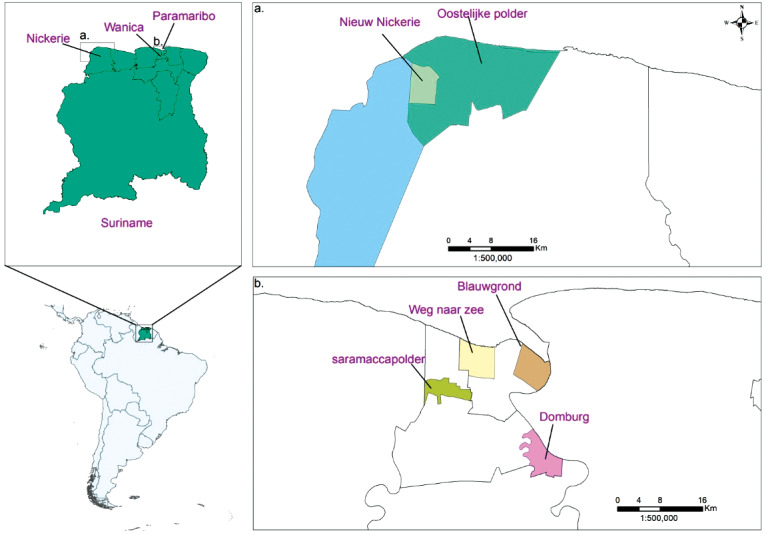
map of the districts of Suriname and the study sites in the selected districts. The map is generated with QGIS version 3.10.10-A Coruña and ArcMap version 10.0.

*Study sites* - In this study we focused on the coastal plain (Fig. 1), where approximately 87 percent of Suriname's population lives.^15^ Six sites were sampled in three districts, Paramaribo, Wanica and Nickerie, where dengue cases are regularly reported. We selected two urban sites at Paramaribo, the capital of Suriname, with the two locations Blauwgrond (5º51'26.523"N, 55º7'9.489"W) and Weg naar Zee (5º52'31.626"N, 55º12'58.984"W), and Wanica with Domburg (5º42'42.005"N, 55º6'3.085"W) and Saramaccapolder (5º49'21.672"N, 55º15'48.265"W) sites. The third district, Nickerie, borders the south of Suriname and is known for its large areas of agriculture, especially the cultivation of rice. Two resorts have sampled there: Nieuw Nickerie (5º39'18.751"N, 56º47'58.097"W) and Oostelijke Polder (5º56'36.957"N, 56º52'32.211"W).

*Mosquito collection and susceptibility test* - From March 2023 through February 2025, fieldwork was conducted in multiple study areas, where a minimum of 30 ovitraps were systematically deployed at each site. Each trap remained in place for a total of eight days, with eggs collected and the trap replaced after four days. The newly placed trap was then collected four days later. The collected eggs were transported to the insectary of the BOG. The eggs per site were carefully pooled and placed in a container with tap water. This ensured the heterogeneity of the population in the samples. After the eggs were left for 2 h in the water, the container was placed in an exicator to de-oxygenise the water to stimulate the breeding of the eggs. After the eggs emerged, the first instar larvae were reared under controlled conditions in the insectary. The larvae were maintained under standard rearing conditions at a temperature of 27 ± 2ºC and a relative humidity of 70 ± 5%. Emerged larvae were fed with fish food till they reached the pupae phase. Pupae were collected with a pipette and placed in a container under a mosquito cage. In every cage, the date was noted as well as other information to make sure we could separate the mosquitoes by age. Adult mosquitoes were fed with a 10% sucrose solution. These adult mosquitoes, aged three to five days, were then tested for insecticide resistance using the Centres for Disease Control and Prevention (CDC) bottle bioassay with 500 mL Wheaton glass bottles. A New Orleans reference strain of *Ae. aegypti* (NO) was also reared under the same conditions and used as a positive control to validate the bioassays. CDC bottle tests were conducted to assess resistance to two insecticides: malathion and λ-cyhalothrin. In accordance with CDC guidelines,^16^ technical-grade malathion (provided by the CDC, USA) was tested at 50 μg/mL and λ-cyhalothrin at 10 μg/mL for all *Ae. aegypti* populations. Per test there were four or five test tubes/badges with an average of 20-25 mosquitoes per tube, and one or two control tubes with the same amount. A synergist bioassay using piperonyl butoxide (PBO) was conducted only for the Saramaccapolder population to evaluate the involvement of oxidase enzymes in resistance mechanisms. Ethanol-treated control bottles were included in all tests for both insecticides. After phenotype testing, a randomly selected number of mosquitoes were preserved frozen at -20ºC, either dry or in RNAlater®.

*Molecular markers associated with resistance* - To investigate to the occurrence of pyrethroid target-site modification mechanism, we screened for three *kdr* mutations (V410L, V1016I and F1534C) already reported worldwide. A subset of adult *Ae. aegypti* mosquitoes collected from three localities [Blauwgrond (20), Weg naar Zee (8), and Nieuw Nickerie (29)] was used for genotyping.

Individual mosquitoes were homogenised in phosphate-buffered saline (PBS), and genomic DNA was extracted using the DNeasy Blood & Tissue Kit (Qiagen, Hilden, Germany), following the manufacturer's protocol. DNA concentrations were measured using a NanoDrop 2000c spectrophotometer (Thermo Scientific, Waltham, MA, USA), and samples were stored at -20 ºC until further analysis.

Genotyping of the V410L (Val/Leu), V1016I (Val/Ile) and F1534C (Phe/Cys) mutations was performed using real-time polymerase chain reaction (PCR) allelic discrimination assays (Applied Biosystems, CA, USA) and the primers, probes, and quantitative PCR (qPCR) cycling conditions followed previously established protocols described in the literature and in a study by Costa et al.^17^ No novel gene sequences were generated in this study.

*Data analysis* - Mosquito populations were classified according to World Health Organisation (WHO) guidelines.^18^ A mortality rate between 98% and 100% indicates susceptibility. When mortality ranges from 90% to 97%, resistance is suspected and must be confirmed through additional bioassays or molecular analyses. If mortality is below 90%, the population is considered resistant, provided that at least 100 mosquitoes were tested. When control mortality is 20% or less, the results can be corrected using Abbott's formula.^19^ The three sites (410, 1016, and 1534) were considered jointly to calculate genotype frequencies. Based on the observed genotypes, the allelic composition was inferred, from which allele frequencies were subsequently derived.^17^ These mutations were analysed jointly given that they belong to the same gene and their physical proximity on the *Ae. aegypti* genome, which suggests they may be in linkage disequilibrium.^20^

*Consent to participate* - Participation in this study was voluntary, and there was no risk to either the participants or their families. Eligible participants were required to be at least 18 years of age, provide informed consent, and the study was explained in Dutch or, if necessary, in their native language by a formal vector control technician, ensuring clarity in communication. The study was conducted in collaboration with the Bureau for Public Health, the local health authority in Suriname.

## RESULTS

**Fig. 2:**
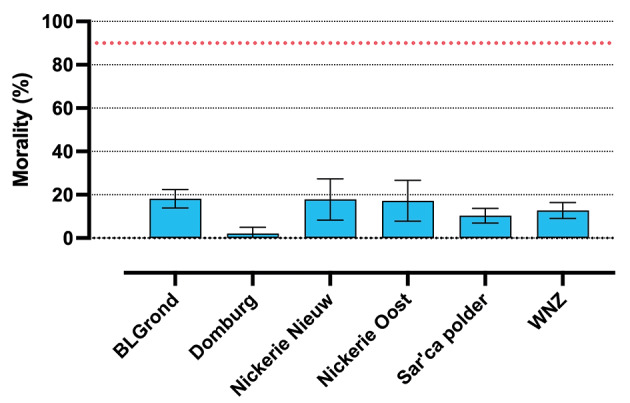
mortality of *Aedes aegypti* populations from Suriname following 30-min exposure to 10 µg λ-cyhalothrin in Centres for Disease Control and Prevention (CDC) bottle bioassays. Bars represent mean mortality, with error bars indicating the standard error of the mean (SEM) for each locality.

**Fig. 3:**
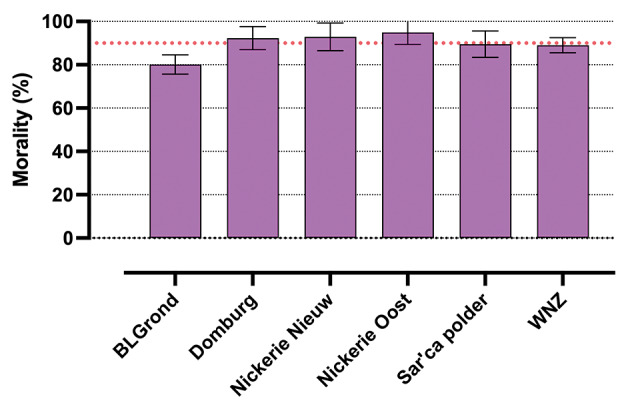
mortality of *Aedes aegypti* populations from Suriname following 30-min exposure to 50 µg malathion in CDC bottle bioassays. Bars represent mean mortality, with error bars indicating the standard error of the mean (SEM) for each locality. The red dotted line indicates the 90% threshold, below which resistance is confirmed.

*Susceptibility bioassays* - During all the tests, there was 100% mortality of mosquitoes of the NO reference strain within 30 min when exposed to the diagnostic dose of malathion and λ-cyhalothrin. Regarding mosquitoes in the negative control group, there was no mortality observed after 30 min of Ethanol exposure. Figs 2-3 show the percentage mortalities of *Ae. aegypti* field strains in six localities when exposed to insecticides λ-cyhalothrin and malathion at the diagnostic dose and time.

For λ-cyhalothrin the mortality rates were all below 20% while the mortality rate for malathion varies between 80.1% and 94.9%

In Saramaccapolder, the mortality rate following exposure to λ-cyhalothrin was 10.3%. In the synergist assay, where the tested mosquitoes were pre-exposed for 1 h to PBO, the mortality rate increased to 43%. Although this represents a significant increase, it nevertheless remains well below the threshold for susceptibility.

**Fig. 4:**
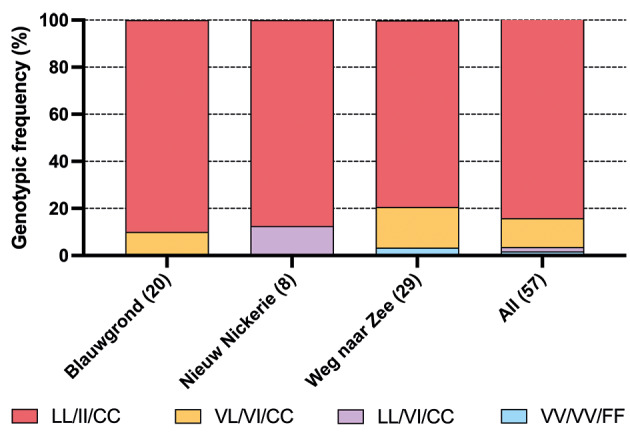
knockdown resistance (*kdr*) genotyping of *Aedes aegypti* populations from Suriname based on combined genotypes at three loci (V410L, V1016I and F1534C) in the voltage-gated sodium channel gene. Bars represent the frequency of tri-locus genotypes in each population. Numbers in parentheses indicate the number of mosquitoes genotyped per location.

*Kdr genotyping* - A total of 57 *Ae. aegypti* mosquitoes were genotyped at three positions associated with *kdr* resistance (410, 1016, and 1534), revealing four tri-locus genotypes. Overall, *kdr* mutations were detected at high frequencies (Table I). The triple homozygous *kdr* genotype leucine/leucine, isoleucine/isoleucine, cysteine/cysteine (LL/II/CC) predominated (84.2%), followed by valine/leucine (VL) / valine/isoleucine (VI) / cysteine/cysteine (CC) (12.3%). The remaining two genotypes, the wild-type valine/valine, valine/valine, phenylalanine/phenylalanine (VV/VV/FF) and leucine/leucine, valine/isoleucine, cysteine/cysteine (LL/VI/CC), together accounted for 3.6% of the total (Fig. 4).

**TABLE I:** Distribution of multilocus genotypes and possible haplotype composition in *Aedes aegypti* from three locations in Suriname

Genotype (410/1016/1534)	Frequency (%)	Possible haplotype composition
LL/II/CC	84.2	LIC / LIC
LL/VI/CC	1.8	LVC / LIC
VL/VI/CC	12.3	VVC / LIC or VIC / LVC*
VV/VV/FF	1.8	VVF / VVF

*For genotype valine/leucine (VL) / valine/isoleucine (VI) / cysteine/cysteine (CC), two alternative haplotype configurations are possible leucine/isoleucine/cysteine (LIC), leucine/valine/cysteine (LVC), valine/valine/cysteine (VVC), and valine/valine/phenylalanine (VVF).

Inferred haplotype frequencies under two alternative phase scenarios are presented in Table II. In both scenarios, the leucine/isoleucine/cysteine (LIC) haplotype was the most frequent.

**TABLE II:** Estimated haplotype frequencies under two alternative scenarios

Haplotype	Scenario 1 (%)^a^	Scenario 2 (%)^b^
LIC	91.2	85.1
VVC	6.1	0.0
LVC	0.9	7.0
VIC	0.0	6.1
VVF	1.8	1.8

*a*: considering the genotype valine/leucine (VL) / valine/isoleucine (VI) / cysteine/cysteine (CC) is composed of the haplotypes valine/valine/cysteine / leucine/isoleucine/cysteine (VVC/LIC); *b*: considering the genotype VL/VI/CC is composed of the haplotypes valine/isoleucine/cysteine / leucine/valine/cysteine (VIC/LVC).

## DISCUSSION

Vector control in Suriname has primarily relied on insecticide-based interventions. As observed globally, such measures may lose effectiveness once reduced susceptibility develops in the target mosquito populations. This study provides the first evidence of malathion resistance in *Ae. aegypti* mosquitoes in Suriname and offers novel insights into the molecular mechanisms underlying this phenomenon. Molecular analysis revealed the presence of individuals carrying the triple-mutant homozygous *kdr* genotype LL/II/CC, strongly associated with pyrethroid resistance, under high frequency. These molecular findings are consistent with the phenotypic assays, which confirmed resistance to λ-cyhalothrin across all six study sites, displaying very low mortality indexes in the bioassays.

The observed resistance to λ-cyhalothrin is notable, given that this insecticide was only introduced by the BOG for vector control in 2019. However, before 2015, other pyrethroids were used, such as deltamethrin, and previous studies have shown that *Ae. aegypti* mosquitoes in different areas of Suriname have developed resistance to deltamethrin.^14^ The results of this study indicate that the mechanisms leading to reduced pyrethroid susceptibility are still strongly present in the population, possibly inducing cross-resistance from deltamethrin to λ-cyhalothrin. This was also noticed in studies in French Guiana, Brazil, and Venezuela.^9^ [Bibr B21] ^21^ In addition, ongoing selection pressure may be reinforced by the widespread use of pyrethroids as pesticides in agriculture, further sustaining this reduced susceptibility phenotype. This was seen in malaria vectors in studies done in Africa.^22^ [Bibr B23] ^23^ Indeed, large quantities of λ-cyhalothrin are imported into Suriname for use as an agricultural pesticide.^24^ Van Sauers-Muller and Ester^25^ reported that only in 2003, 19.052 kg/L λ-cyhalothrin were registered as imported in Suriname. Data from the Department of Pesticides of the Ministry of Agriculture, Livestock, and Fisheries in Suriname showed that between 2019 and 2022, the average annual import of λ-cyhalothrin was 61,523 litres/year (Unpublished data). Of note, the import of λ-cyhalothrin has been tripled over the past 10 years, suggesting an increasing usage. This keeps a high selection pressure in favour of certain resistance mechanisms, which is manifested by high levels of resistance despite moderate use of the insecticide by BOG. Dusfour et al.^26^ mention a great loss of the effectiveness of pyrethroids in three French overseas countries, including French Guiana. In Brazil and Argentina, studies mentioned widespread pyrethroid resistance.^27^ [Bibr B28] ^28^ [Bibr B29] ^29^ The results of this study show that *Ae. aegypti* in Suriname is also resistant to pyrethroids, and this must be considered in the national vector control programs.

In Saramaccapolder, the mortality rate increased when the mosquitoes were pre-exposed to PBO before the testing with λ-cyhalothrin, although it was still resistant. This suggests that, beyond *kdr* mutation events, metabolic enzymes, such as monooxygenases P450, also play a key role in the resistance.

At Blauwgrond, the result of 80.1% mortality shows resistance to malathion. As far as we know, this is the first confirmed case of malathion resistance in Suriname. On Weg naar Zee, the result showed a 89% mortality rate, which indicates reduced susceptibility. Saramaccapolder had a mortality rate of 88.9% while the other areas showed mortality between 90-97%. The results are an indication of a decline in malathion effectiveness. In none of the cases was susceptibility observed to malathion, which is alarming because past tests till 2017^14^ indicated 98-100% susceptibility to malathion in different areas of Suriname. Changes in the susceptibility of *Ae. aegypti* to malathion have already been documented in several Latin-American countries, including Brazil, Venezuela, and Jamaica, although the results among the regions differ.^26^ [Bibr B5] ^5^ [Bibr B21] ^21^ [Bibr B10] ^10^ Since malathion has so far been used in vector control in Suriname and resistance has now been observed for the first time, it calls for alertness from the authorities. Multiple areas should be investigated for the resistance status of the *Ae. aegypti*. As for pyrethroids, it is also possible that the selection pressure for resistance to malathion is increased by their use in agricultural pesticides. Indeed, and as for malathion, 19,004 L were registered as imported in Suriname only in 2003.^23^ Similarly, data from the Department of Pesticides of the Ministry of Agriculture, Livestock, and Fisheries in Suriname showed that between 2019 and 2022, the average annual import of malathion was 23,006 litres/year (Unpublished data). This maintained a strong selection pressure for resistance in vector populations.

Molecular analysis conducted as part of this study represents a pioneering effort in the understanding of insecticide resistance mechanisms in *Ae. aegypti* populations in Suriname. Although the number of samples remains limited, this study is particularly critical given that it is the first of its kind to identify resistance mutations within the country. The triple *kdr* homozygous genotype (LL/II/CC), which confer a higher selective advantage under insecticide pressure, was the most observed. Remarkably, the wild-type genotype VV/VV/FF was found only in one sample. The levels and combinations of the multilocus genotypes and haplotypes are close to those found in the *Ae. aegypti* populations in French Guiana, Venezuela, and the neighbouring regions of Brazil, although previous studies have shown that genotype and allele frequency compositions may vary significantly by geographic location.^9^ [Bibr B21] ^21^ [Bibr B17] ^17^ [Bibr B30] ^30^ The high frequencies of the triple *kdr* homozygous genotype reported in this study should be interpreted in the context of the study design. Although genotype frequency data from multiple localities were pooled for presentation to provide a country-level overview of resistance, samples from each area were genotyped independently. Notably, the triple *kdr* homozygous genotype was consistently observed at high frequency across all sampled areas, but the limited number of specimens per area did not allow statistically reliable, area-specific estimates of genotype frequencies. Nevertheless, given the high level of target-site resistance observed in this study and the frequent co-occurrence of multiple resistance mutations in individual mosquitoes, monitoring resistance levels and underlying mechanisms across different regions would be highly valuable.

*In conclusion* - The outcomes of this study mark an important step forward in our view and comprehension of (reduced) insecticide susceptibility in *Ae. aegypti* in Suriname. As the first molecular findings of *kdr* mutations, partially explaining the well-established decreased sensitivity to pyrethroids, and the first case of malathion resistance in this country, they underline the importance of establishing a foundation for further surveillance and systematic monitoring of resistance in vector populations, including genetic analyses. The combination of bioassay results and *kdr* mutation frequencies in natural populations offers essential insights into resistance intensity and contributes to the design of effective vector control strategies and evidence-based public health policies.

## Data Availability

The contents underlying the research text are included in the manuscript.
