# Supplementary material for: Evidence of insecticide resistance in Aedes aegypti populations of Suriname: first report of malathion resistance and presence of the kdr mutations V410L, V1016I and F1534C
Source: Mem Inst Oswaldo Cruz. 2026 Jul 10;121:e250335. doi: 10.1590/0074-02760250335 (PMC13361133; doi:10.1590/0074-02760250335)
Supplement: Peer review history

# Reviewer report: FIRST REVIEW ROUND - REVIEWERS' COMMENTS

## REVIEWER #1

General Comments

The manuscript presents valuable data on insecticide resistance and *kdr* mutations in *Aedes aegypti* from Suriname, a region for which limited information is currently available. The study clearly reflects a significant experimental and logistical effort, and the data generated have the potential to make a meaningful contribution to the regional literature on insecticide resistance.

Nevertheless, some aspects related to nomenclature consistency, clarity of methodological descriptions, and data presentation require revision in order to strengthen the manuscript and improve its readability and scientific accuracy. The specific comments below are intended to be constructive and to help improve the overall quality of the work.

Specific Comments

1. Nomenclature of *kdr* mutations. In general, *kdr* mutations are named according to their position along the voltage-gated sodium channel. I therefore recommend using a consistent order throughout the manuscript: 410, 1016, and 1534. Please revise the entire manuscript, including figures and tables.

2. Keywords. I suggest remove the word populations from *Ae aegypti* populations and use simply *Ae aegypti*.

3. Disease names. In the Introduction (first sentence) and throughout the manuscript, please use capitalization only for Zika, while other disease names should remain in lowercase, ensuring consistency across the text.

4. Capitalization of insecticides and solvents. Names of insecticides and solvents should be written in lowercase. For example (page 5, line 8): organophosphates, including malathion, fenitrothion, and pyrethroids such as deltamethrin and cypermethrin. Also, ethanol; piperonyl butoxide, and more. Please revise accordingly.

5. Species abbreviation. Page 3, line 8: replace *Aedes aegypti* with *Ae aegypti*. Please apply this abbreviation consistently throughout the manuscript after the first full mention.

6. Synergists definition. Page 3, line 40: "Synergists are chemicals that inhibit these enzymes in mosquitoes and are used in insecticides to enhance their potency" I believe that should be "Synergists are chemicals that inhibit these enzymes in mosquitoes and are used in combination with insecticides to enhance their potency".

7. Mode of action of pyrethroids. Page 3, line 47: The sentence states that pyrethroids were "developed" to act at the voltage-gated sodium channel. This is not accurate. Please revise to indicate that this channel is the target site at which pyrethroids act, rather than implying intentional development for this site.

8. Population description. Page 4, lines 53–54: the sentence "In this study we focused on the coastal plain (Figure 1), where approximately 87 percent of Suriname's population of …" appears to be incomplete or unclear. Please revise and clarify what is being referred to.

9. Rearing conditions and protocols. Page 5, lines 44–45: Please specify in which institution the insectary is located. Additionally, clarify which standard laboratory protocols were followed or provide appropriate references.

10. Order of *kdr* mutations in results. Page 6, lines 28–29 and 52–53: Please reorder the *kdr* mutations consistently as 410, 1016, and 1534.

11. Data analysis redundancy. Page 7, lines 53–54: The description of allele frequency calculation using the formula (2p + pq)/2n has already been presented earlier (lines 11–20). Please avoid repetition or refer back to the previous description.

12. Figures 2 and 3: variability and error measures. Figures 2 and 3 do not show variability. Please indicate whether standard error or standard deviation was calculated and include this information in the figures or figure legends.

13. Genotype nomenclature and clarity. Page 9, lines 9–15: Because *kdr* mutations are named in different orders throughout the manuscript, it is unclear which SNP each genotype refers to (e.g., LL/II/CC, VL/VI/CC). This issue also affects Figure 5. Please standardize mutation order and clearly indicate which positions the letters refer to in the genotype notation.

14. Allele frequency clarification. Page 9, lines 3–5: When stating that the frequency of the resistant 410L, 1016I, and 1534C alleles is 0.92, 0.91, and 0.98, respectively, please clarify whether these correspond to the Leu, Ile, and Cys alleles.

15. Discussion: interpretation of pooled samples. In the discussion, high frequencies of *kdr* mutations and the triple homozygous resistant genotype are emphasized. It is important to clearly state that the genotyped mosquitoes represent a pool from different localities. While this provides a general overview, it may mask local variation. Please clarify this in the discussion.

## REVIEWER #2

This study is very well written and presented. It is relevant because it is the first study in Suriname to report resistance to the organophosphate insecticide malathion and the pyrethroid lambda-cyhalothrin in *Aedes aegypti* populations. Furthermore, it provides evidence that *kdr* mutations contribute to pyrethroid resistance. This study fills an important gap in our understanding of resistance in this region.

The methodology used was based on the CDC bottle bioassay, which is supported by the World Health Organization. Likewise, the references used are relevant, as they include an adequate review of the scientific literature related to *kdr* mutations worldwide.

## AUTHORS' RESPONSE TO THE REVIEWERS

**REVIEWER COMMENTS:**

Reviewer: 1

General Comments

The manuscript presents valuable data on insecticide resistance and *kdr* mutations in *Aedes aegypti* from Suriname, a region for which limited information is currently available. The study clearly reflects a significant experimental and logistical effort, and the data generated have the potential to make a meaningful contribution to the regional literature on insecticide resistance.

Nevertheless, some aspects related to nomenclature consistency, clarity of methodological descriptions, and data presentation require revision in order to strengthen the manuscript and improve its readability and scientific accuracy. The specific comments below are intended to be constructive and to help improve the overall quality of the work.

Specific Comments

1. We agree with the reviewer and have revised the entire manuscript to ensure that *kdr* mutations are consistently presented in the order 410, 1016, and 1534. This change has been applied throughout the text, as well as in all tables and figures.

2. We agree with the reviewer and have revised the keywords accordingly by removing the word "populations" and using *Ae aegypti*.

3. We agree with the reviewer and have revised the manuscript to ensure that all insecticide and solvent names are consistently written in lowercase throughout the text.

4. We agree with the reviewer and have revised the manuscript to ensure that all insecticide and solvent names are consistently written in lowercase throughout the text.

5. We agree and have revised the manuscript so that *Aedes aegypti* is abbreviated as *Ae aegypti* after the first full mention, and this abbreviation is now used consistently throughout the manuscript.

6. We agree with the reviewer and have replaced the original sentence with the suggested wording to clarify that synergists are used in combination with insecticides to enhance their potency (page 3, line 40).

7. We agree with the reviewer and have revised the sentence to clarify that voltage-gated sodium channels are the target site at which pyrethroids act, rather than implying intentional development for this site (page 3, line 47).

8. We agree and have revised the sentence to improve clarity. The sentence has been completed and clarified to explicitly state that approximately 87% of Suriname's population lives in the coastal plain (page 4, lines 53–54).

9. We have revised the manuscript to specify that the insectary is located at the Entomology Department of the Bureau of Public Health (BOG). In addition, we have expanded the description of the egg hatching and rearing procedures in the section Mosquito collection and susceptibility test to provide more detail on the standard protocols followed.

10. We agree with the reviewer and have revised the Results section to ensure that *kdr* mutations are consistently presented in the order 410, 1016, and 1534.

11. We agree and have revised the manuscript to remove the redundancy. The allele frequency formula is now presented only once (page 7, lines 11–20), and the repeated description has been deleted.

12. We agree and put the SD proportion in the figures 2 and 3

13. We agree with the reviewer that genotype notation must be fully unambiguous. Although the mutation order was indicated in figure 5, we have revised the results section to explicitly state which alleles correspond to positions 410, 1016, and 1534. This clarification ensures that genotype codes such as LL/II/CC are unambiguously interpretable.

14. We agree with the reviewer and have revised the text to explicitly clarify that the reported allele frequencies correspond to the leucine (410L), isoleucine (1016I), and cysteine (1534C) resistant alleles (page 9, lines 3–5).

15. We agree with the reviewer that it is important to clarify the interpretation of results derived from pooled samples, but want to give some clarification:

The presence of the triple homozygous resistant genotype is indeed emphasized in the discussion, and we acknowledge that the genotyped mosquitoes represent a pooled dataset originating from multiple localities. Importantly, genotyping and initial analyses were performed at the area level, and the triple homozygous resistant genotype was consistently observed at high frequency across all sampled areas. While pooling data for a country-level overview may limit area-specific interpretation, the consistent presence of LL/II/CC across all areas reinforces the overall conclusion regarding widespread *kdr*-mediated resistance in Suriname. We have revised the discussion to clarify that the results should be interpreted as a general country-level overview, and that the limited number of specimens per area did not allow statistically reliable area-specific frequency estimates.

Reviewer: 2

This study is very well written and presented. It is relevant because it is the first study in Suriname to report resistance to the organophosphate insecticide malathion and the pyrethroid lambda-cyhalothrin in *Aedes aegypti* populations. Furthermore, it provides evidence that *kdr* mutations contribute to pyrethroid resistance. This study fills an important gap in our understanding of resistance in this region.

The methodology used was based on the CDC bottle bioassay, which is supported by the World Health Organization. Likewise, the references used are relevant, as they include an adequate review of the scientific literature related to *kdr* mutations worldwide.

Response to Reviewer 2:

We sincerely thank the reviewer for the very positive and encouraging evaluation of our manuscript, and for recognizing the relevance of the study and the appropriateness of the methodology and references used. Since no specific revisions were requested, no changes were made to the manuscript in response to this reviewer.

Note: For ease of review, all changes made in response to the reviewers' comments are highlighted in red. In addition, a small number of minor, non-substantive changes were made following an internal review by the authors; these do not affect the results or conclusions. All changes are shown in red.

# Reviewer report: HANDLING EDITOR COMMENTS

## HANDLING EDITOR COMMENTS

I appreciate the effort invested in addressing the points previously raised.

I have now conducted a thorough reading of the revised manuscript and inserted my additional comments directly into the PDF file for your consideration.

At this stage, my main concerns are the analysis and presentation of the *kdr* genotyping data. As currently structured, the analyses still treat each SNP independently. However, since the three mutations investigated (V410L, V1016I, and F1534C) are located within the same gene and were genotyped in the same individuals, it is more informative and biologically meaningful to analyse them jointly as multilocus genotypes.

I strongly require that you reanalyze the data accordingly. Specifically, you should:

(1) list (and plot) all observed combined multilocus genotypes/ population (and or pooled for Suriname);

(2) calculate multilocus genotype frequencies for each population; and

(3) infer the corresponding alleles/haplotypes and estimate their frequencies when appropriate.

Analyzing the loci separately overlooks the biological and evolutionary relevance of specific mutation combinations, which are often the true operational markers under selection. Presenting the data at the multilocus genotype level will substantially strengthen the evolutionary interpretation and scientific value of this study.

I look forward to receiving a revised version addressing these points.

## AUTHORS' RESPONSE TO THE REVIEWERS

I strongly require that you reanalyze the data accordingly. Specifically, you should:

(1) list (and plot) all observed combined multilocus genotypes/ population (and or pooled for Suriname) We agree and revised figure 5. All observed multilocus genotypes per location and pooled are presented

(2) calculate multilocus genotype frequencies for each population; and infer the corresponding alleles/haplotypes and estimate their frequencies when appropriate.

We understand and add this information in 2 tables mentioned in page 5 line 8-12

Multilocus genotype distributions are shown in Table I. The fully resistant genotype LL/II/CC predominated (84.2%), followed by VL/VI/CC (12.3%), while other genotypes were rare.

Inferred haplotype frequencies under two alternative scenarios are presented in Table II. In both scenarios, the LIC haplotype was the most frequent.

# Reviewer report: HANDLING EDITOR COMMENTS

## HANDLING EDITOR COMMENTS

Corrections indicated by the handling editor in the attached PDF. See the link below:

https://memorias.ioc.fiocruz.br/images/revistas/2025/open-review/25-0335-open-review.pdf

## AUTHORS' RESPONSE TO THE REVIEWERS

Dear Editor and Reviewers,

We would like to sincerely thank you for your thorough evaluation of our manuscript and for the constructive comments provided. We have carefully considered all remarks and have revised the manuscript accordingly.

All changes have been clearly indicated in red in the revised version to facilitate review.

In response to the comments regarding the analysis and presentation of the *kdr* genotyping data, we have substantially revised this section. Table 4 has been replaced with a new table providing more detailed information on the molecular analyses. In addition, two new tables have been included to present the multilocus genotype distributions (Table I) and inferred haplotype frequencies under two alternative phase scenarios (Table II).

We believe that these revisions have significantly strengthened the manuscript and improved the clarity and scientific value of our findings.

We hope that the revised version meets the journal's requirements and we look forward to your decision.

Sincerely,

Kartika Doerdjan-Ramoutar MSc.

# Reviewer report: HANDLING EDITOR COMMENTS

## HANDLING EDITOR COMMENTS

No comments
